# Right ventricular dysfunction: an overlooked predictor of sudden cardiac death and arrhythmic events—a meta-analysis

**DOI:** 10.1007/s00392-026-02976-6

**Published:** 2026-07-02

**Authors:** Toshinori Chiba, Amelie Beblo, Nora Wainstejn, Julia Lueg, Robert Hättasch, Felix Hohendanner, Verena Tscholl, Nikolaos Dagres, Gerhard Hindricks, Wilhelm Haverkamp

**Affiliations:** Department of Cardiology, Deutsches Herzzentrum Der Charité, Campus Mitte, Berlin, Germany

**Keywords:** Right ventricular dysfunction, Sudden cardiac death, Ventricular arrhythmia, Implantable cardioverter-defibrillator

## Abstract

**Background:**

Right ventricular (RV) dysfunction has emerged as a potential predictor of adverse cardiac outcomes, including sudden cardiac death (SCD) and severe ventricular arrhythmias (VAs). To clarify the role of RV dysfunction in arrhythmic risk assessment, we conducted a systematic review and meta-analysis to evaluate its prognostic value for SCD and VA.

**Methods:**

A systematic search of PubMed, Embase, and Web of Science was conducted from inception to February 2025. The primary endpoint was a composite of SCD or severe VA, including ventricular tachycardia, ventricular fibrillation, or appropriate implantable cardioverter-defibrillator therapy. RV dysfunction was defined as impaired RV fractional area change, RV ejection fraction, or RV strain. A random-effects model was used to calculate pooled odds ratios (ORs) and mean differences (MDs) with 95% confidence intervals (CIs).

**Results:**

Of 1296 identified articles, seven studies comprising 1475 patients met the inclusion criteria. RV dysfunction was present in 541 patients. The mean follow-up duration was 2.9 years. RV dysfunction was significantly associated with an increased risk of SCD and VA (OR 3.69; 95% CI 2.40–5.66; *p* < 0.01). Secondary analysis showed that patients who experienced SCD or VA had significantly lower RVFAC compared to those without events (MD − 5.67; 95% CI − 8.73 to − 2.60; *p* < 0.01), whereas LVEF did not differ significantly between groups (MD − 0.59; 95% CI − 4.10 to 2.92; *p* = 0.74).

**Conclusions:**

RV dysfunction is associated with an increased risk of SCD and VA, indicating that RV parameters may hold prognostic value in arrhythmic risk stratification.

**Graphical Abstract:**

**Figure:**
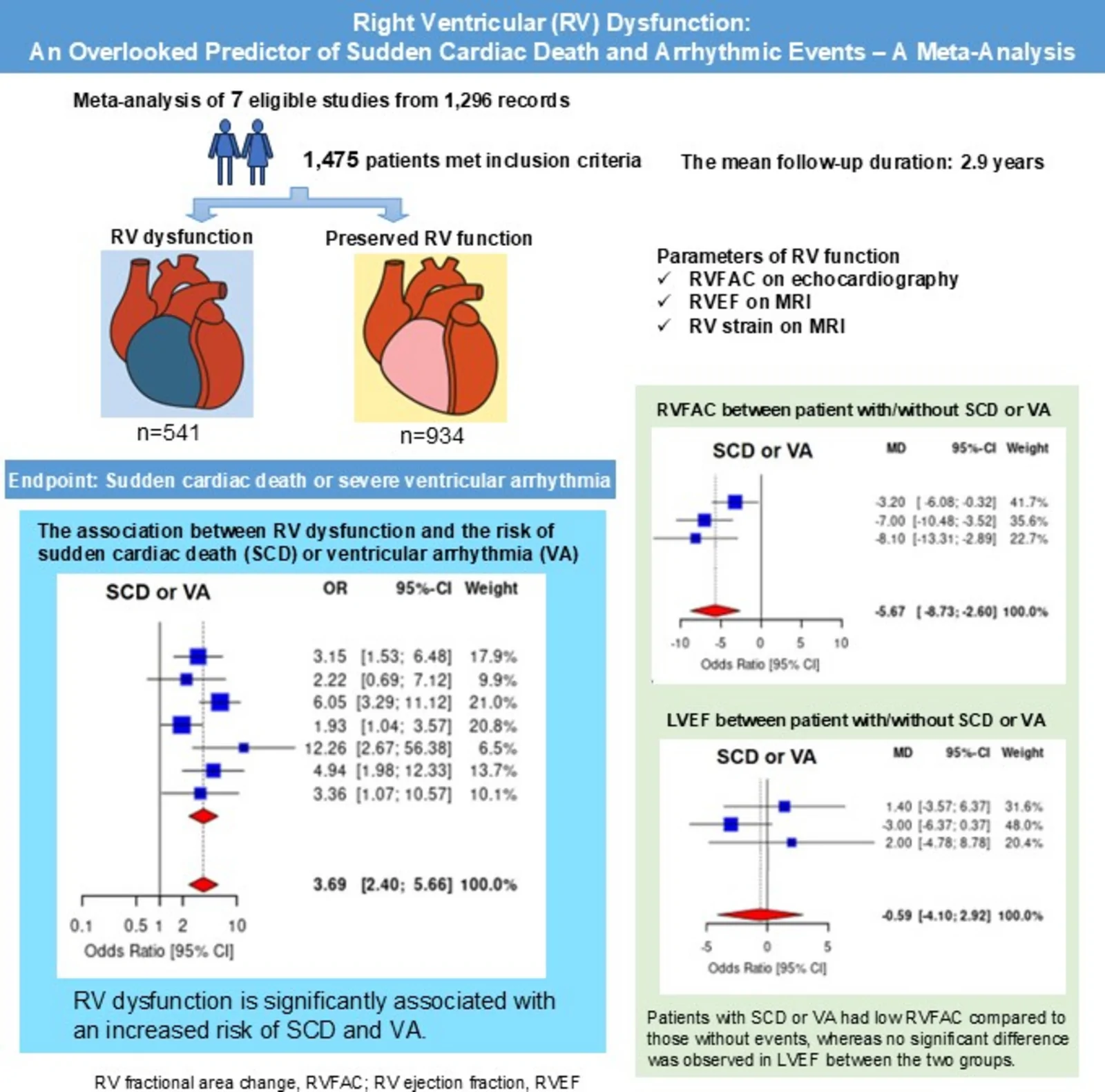

**Supplementary Information:**

The online version contains supplementary material available at https://doi.org/10.1007/s00392-026-02976-6.

## Introduction

Sudden cardiac death (SCD) remains a major public health concern, with an estimated global incidence ranging from 30 to 100 per 10,000 individuals annually [1, 2]. Despite advances in emergency response systems—such as automated external defibrillators and increased rates of bystander cardiopulmonary resuscitation—ventricular arrhythmias (VAs), particularly ventricular tachycardia (VT) and ventricular fibrillation (VF), remain the predominant causes of out-of-hospital cardiac arrests [3].

Left ventricular (LV) dysfunction, especially a reduced LV ejection fraction (LVEF), is a well-established risk factor for SCD and underpins current guideline recommendations for implantable cardioverter-defibrillator (ICD) implantation in primary prevention. Patients with an LVEF ≤ 35% are considered at high risk and are eligible for ICD therapy, which has been shown to reduce mortality by preventing life-threatening arrhythmias [4]. Accordingly, both American and European guidelines (AHA/ACC/HRS and ESC) emphasize LVEF as a central criterion for ICD implantation [5, 6].

However, growing evidence suggests that reliance on LVEF alone may be inadequate for comprehensive risk stratification. Notably, Stecker et al. reported that only approximately one-third of SCD cases occur in patients with an LVEF ≤ 35%, while nearly half occur in individuals with preserved LV function [7]. This discrepancy underscores the need for additional, more sensitive predictors of arrhythmic risk.

Emerging data point to the prognostic significance of right ventricular (RV) dysfunction in this context. RV impairment has been associated with adverse outcomes in a range of cardiac conditions, including heart failure progression and increased susceptibility to VAs. Several studies, including those by Pandat et al. and our own group, have demonstrated that reduced right ventricular fractional area change (RVFAC) is independently associated with an increased risk of SCD or appropriate ICD therapy [8, 9]. Moreover, Elming et al. observed that among patients with LV dysfunction, coexisting RV dysfunction was linked to a greater survival benefit from ICD implantation, further suggesting a role for RV function in refining risk stratification [10].

In light of these findings, we conducted a systematic review and meta-analysis to evaluate the association between RV dysfunction and the risk of SCD and VA. We also sought to compare the prognostic value of RV function with LVEF to assess whether RV parameters may offer incremental benefit in arrhythmic risk prediction.

## Methods

This meta-analysis was conducted in accordance with the Preferred Reporting Items for Systematic Reviews and Meta-Analyses (PRISMA) guidelines [11]. The study protocol was prospectively registered in the PROSPERO database on 14 May 2025 (registration number CRD420251045088).

### Eligibility criteria

Studies were eligible for inclusion if they met the following criteria: (1) observational cohort studies (prospective or retrospective) or randomized controlled trials; (2) investigated the association between RV dysfunction and SCD or VA events; and (3) provided extractable data for risk estimation (e.g., number of events, hazard ratios, odds ratios, or risk ratios), included a minimum follow-up period of at least 12 months, and assessed RV function using echocardiography or cardiac magnetic resonance imaging (MRI).

We excluded case reports, editorials, letters, conference abstracts, systematic reviews, meta-analyses, and studies without extractable outcome data. A secondary objective was to compare the prognostic utility of RV dysfunction with that of LVEF in predicting SCD or VA.

### Data sources and search strategy

A comprehensive literature search was performed using PubMed, Embase, and Web of Science from database inception to February 2025. Search terms included.

(“right ventricular dysfunction” OR “right ventricular function”) AND (“sudden death” OR “ventricular arrhythmia” OR “ICD” OR “implantable cardioverter defibrillator”).

Two reviewers independently screened all titles and abstracts, followed by full-text review. Disagreements were resolved by discussion or consultation with a third reviewer. Reference lists of all eligible articles were manually searched for additional studies.

### Data extraction

Data were extracted using a standardized form and included study characteristics (first author, publication year, and study design), patient demographics (sample size, age, sex distribution, comorbidities, and clinical background), RV functional parameters (RVFAC, RV ejection fraction (RVEF), and RV strain), primary outcomes (SCD, VA, and appropriate ICD therapy (shock or antitachycardia pacing)), and follow-up duration.

### Endpoint definitions

The primary endpoint was a composite of SCD or VA, defined as SCD or aborted SCD (documented VT/VF, resuscitated cardiac arrest), and appropriate ICD therapy (shock or antitachycardia pacing). These endpoints were collectively referred to as “SCD or VA events” for analysis.

### Statistical analysis

Statistical analyses were performed using R software version 4.4.3 (R Foundation for Statistical Computing, Vienna, Austria) and followed the Cochrane Handbook for Systematic Reviews of Interventions [12]. Dichotomous outcomes were pooled using random-effects models to calculate pooled odds ratios (ORs) with 95% confidence intervals (CIs). Continuous outcomes were analyzed using inverse-variance random-effects models to estimate mean differences (MDs).

If necessary, missing event numbers were estimated using the method of Tierney et al., which allows for calculation of log hazard ratios from published data [13]. For studies reporting medians and interquartile ranges, means and standard deviations were approximated using the method of Luo et al. [14].

Heterogeneity was assessed using Cochran’s *Q* test and the *I*^2^ statistic. Sensitivity analyses were conducted to examine the impact of data estimation methods. A secondary analysis was performed to compare the prognostic relevance of RV versus LV function.

Study quality was evaluated using the Newcastle–Ottawa Scale [15, 16]. Publication bias was assessed using funnel plots and Egger’s regression test, with *p* values < 0.05 indicating potential bias. A *p* value < 0.05 was considered statistically significant throughout.

## Results

### Study selection

**Fig. 1:**
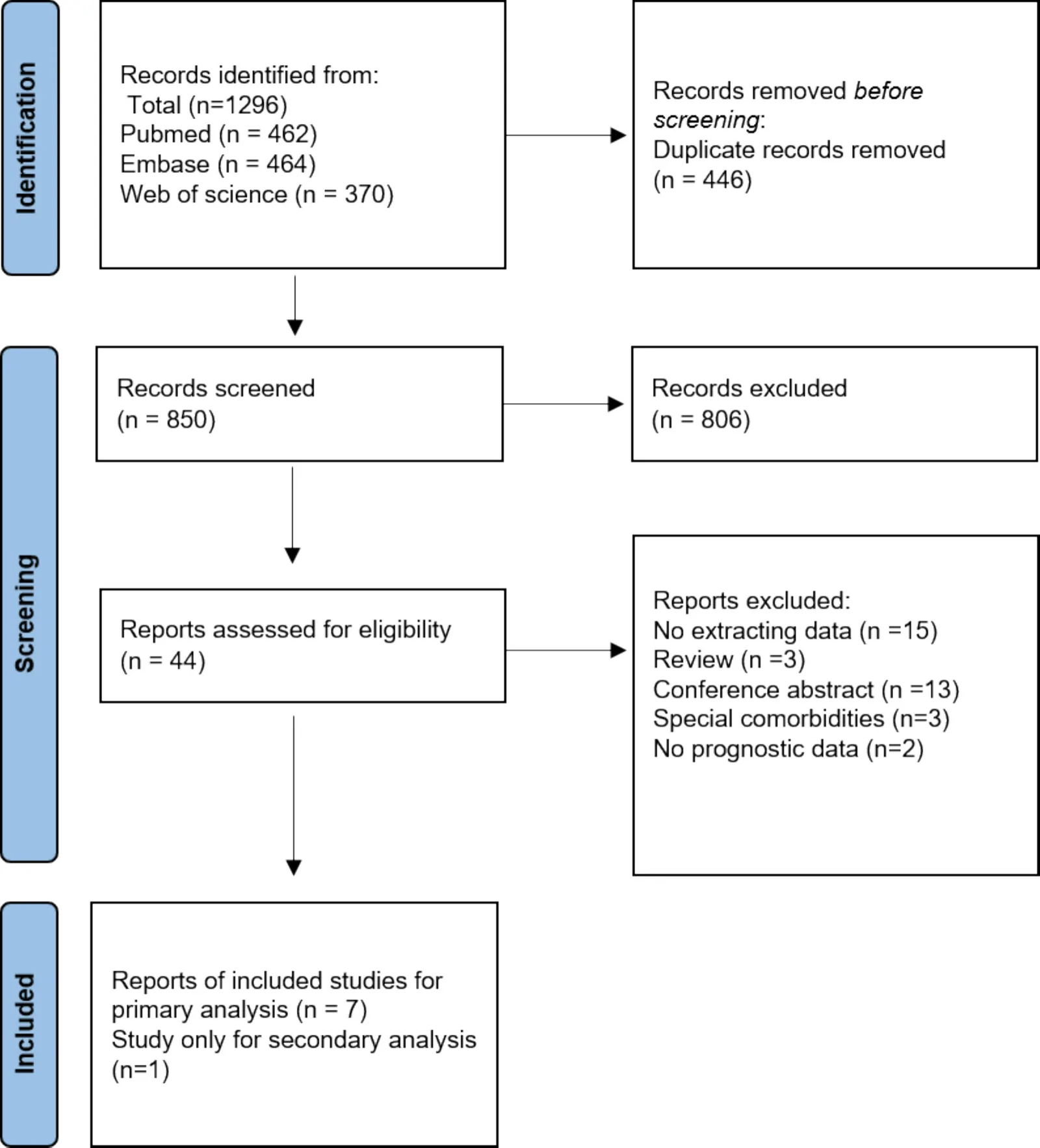
Flowchart illustrating the study selection process

A total of 1296 records were identified through database searches: 462 from PubMed, 464 from Embase, and 370 from Web of Science. After removal of 446 duplicates, 850 records remained for title and abstract screening. Of these, 806 were excluded as irrelevant to the study objective. The full texts of 44 articles were assessed for eligibility, and 7 studies [8–10, 17–20] met the inclusion criteria for the primary meta-analysis. One additional study [21] was included solely for secondary analysis. The study selection process is illustrated in the PRISMA flowchart (Fig. 1).

### Study characteristics

Table 1 summarizes the characteristics of the included studies. Studies 1 through 7 were included in the primary meta-analysis. Study 8 was used exclusively for the secondary analysis, as it stratified patients based on appropriate ICD shocks, making extraction of RV dysfunction-specific outcome data infeasible.

**Table 1:** Study characteristics

Study number	First author [reference]	Year	Country	Sample size	Observational period	Endpoint	Study design	Age	Male	Study population	Definition of RV dysfunction
1	Chiba [9]	2024	Japan	172	4.7 years	ICD therapy^*^	Retrospective	60.3 ± 13.6	76.2%	ICD patients	RVFAC < 34.8%
2	Elming [10]	2019	Denmark	128	5.3 years	SCD^†^	Prospective	61	73%	Nonischemic cardiomyopathy (LVEF ≤ 35%)	RVEF ≤ 45%
3	Pandat [8]	2019	USA	350	1.0 year	SCD^‡^	Prospective	68.7 ± 13.6	73%	No specific	RVFAC ≤ 35%
4	Mikami [17]	2017	Canada	314	2.1 years	SCD^§^ or ICD therapy^*^	Prospective	59 ± 13	73.6%	Ischemic or nonischemic dilated cardiomyopathy (LVEF ≤ 54%)	RVEF ≤ 45%
5	Becker [18]	2021	Netherlands	168	2.2 years	VA^¶^	Retrospective	58 ± 14	42%	Nonischemic cardiomyopathy (LVEF < 50%)	RVEF < 45%
6	Pfisterer [19]	1986	Switzerland	127	1.0 year	SCD^§^ or VA^‖^	Prospective	59	81%	Myocardial infarction	RVEF ≤ 35%
7	Elming [20]	2023	Denmark	216	5.7 years	SCD^†^	Prospective	63.5	72.6%	Nonischemic cardiomyopathy (LVEF ≤ 35%)	RV-FWS > −20%
8	Malasana [21]	2011	USA	57	2.6 years	ICD shock therapy	Retrospective	66.0	82.5%	ICD patients	-

*ICD* implantable cardioverter-defibrillator, *LVEF* left ventricular ejection fraction, *RVFAC* right ventricular fractional area change, *RVEF* right ventricular ejection fraction, *RV-FWS* right ventricular free wall strain, *SCD* sudden cardiac death, *VA* ventricular arrhythmia

^*^Appropriate ICD therapy was defined as either appropriate shock therapy or antitachycardia pacing

^†^SCD was defined as death occurring unexpectedly in a previously stable patient, death occurring within 1 h of symptom onset or worsening, or unwitnessed death in a patient last seen alive within 72 h before death without evidence of life-threatening disease or symptoms, when the circumstances were consistent with sudden death

^‡^SCD was defined as a sudden and unexpected pulseless condition of likely cardiac etiology when witnessed or as sudden death within 24 h of the patient last being seen in a usual state of health when unwitnessed

^§^SCD was defined as death occurring within 1 h of symptom onset

^¶^VA was defined as resuscitated cardiac arrest, sustained ventricular tachycardia, ventricular fibrillation, or appropriate ICD therapy

^‖^VA was defined as Lown class IV ventricular arrhythmias

A total of 1475 patients were included, of whom 541 had RV dysfunction. The mean follow-up duration across studies was 2.9 years. RV function was assessed using RVFAC in two studies and RVEF in four studies; one study employed RV free wall strain. Five of the seven primary studies had a prospective design. Study populations varied: one was community-based (a registry of SCD), and four included patients with ischemic or nonischemic cardiomyopathy. Detailed definitions of the endpoint components for each included study are summarized in Table 1.

Quality assessment using the Newcastle–Ottawa Scale (Table 2) showed that studies scored between 6 and 9 points, with a mean score of 7.9. Most studies were classified as high quality; only one was considered of moderate quality. Egger’s regression test indicated no significant publication bias (*p* = 0.58).

**Table 2:** Quality assessment using the Newcastle–Ottawa Scale

Study	Representativeness of the exposed cohort (0–1)	Selection of the nonexposed cohort (0–1)	Ascertainment of exposure (0–1)	Outcome not present at start (0–1)	Comparability of cohorts (0–2)	Assessment of outcome (0–1)	Follow-up long enough for outcomes to occur (0–1)	Adequacy of follow-up of cohorts (0–1)	Total score (0–9)	Overall quality
Chiba et al. (2024)	1	0	1	1	2	1	1	1	8	High
Elming et al. (2019)	1	1	1	1	2	1	1	1	9	High
Pandat et al. (2019)	1	1	1	1	1	1	1	1	8	High
Mikami et al. (2017)	1	0	1	1	2	1	1	1	8	High
Becker et al. (2021)	1	0	1	1	2	1	1	0	7	High
Pfisterer et al. (1986)	1	0	1	1	0	1	1	1	6	Moderate
Elming et al. (2023)	1	1	1	1	2	1	1	1	9	High

### RV dysfunction and risk of sudden cardiac death or ventricular arrhythmia

**Fig. 2:**
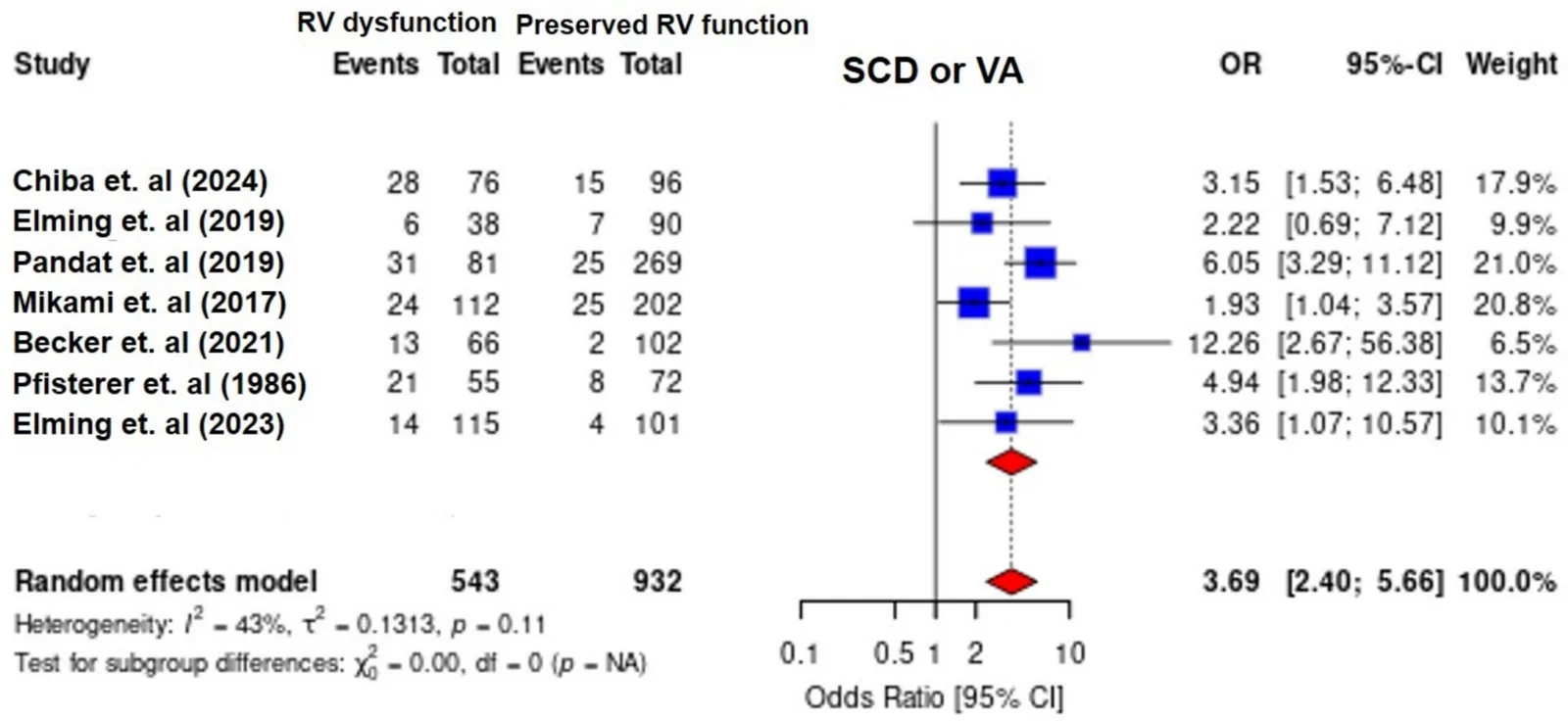
Forest plot illustrating the association between right ventricular dysfunction and the risk of sudden cardiac death or ventricular arrhythmias. RV dysfunction was significantly associated with an increased risk of sudden cardiac death or ventricular arrhythmias, with a pooled odds ratio (OR) of 3.69 (95% CI 2.40–5.66) and moderate heterogeneity (*I*^2^ = 43%). RV, right ventricular; SCD, sudden cardiac death; VA, ventricular arrhythmia

**Fig. 3:**
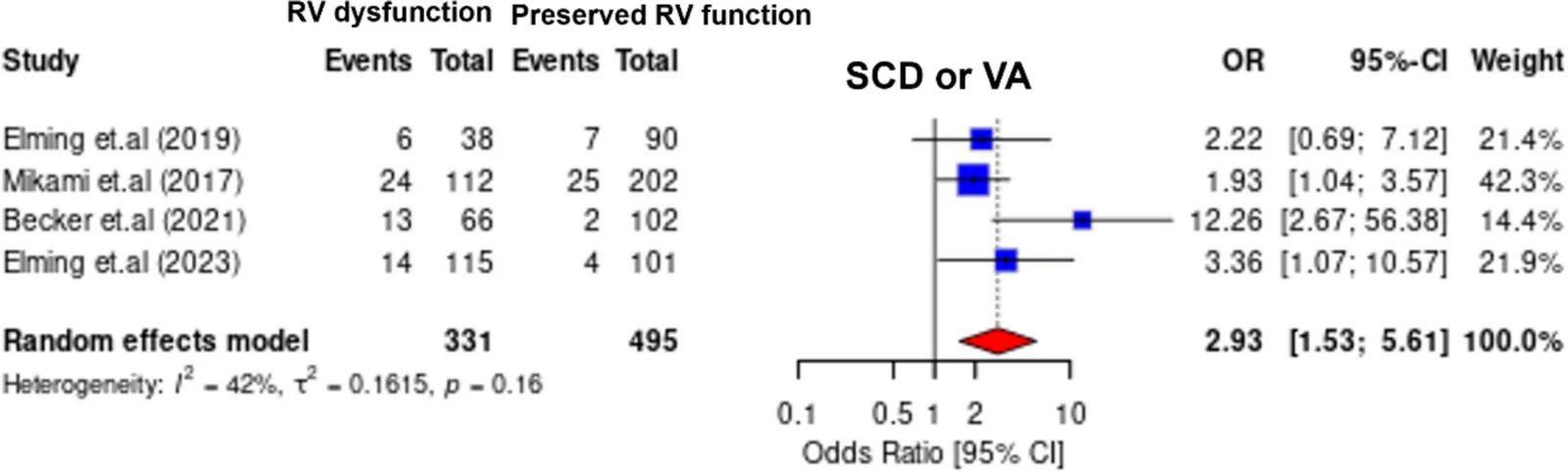
Forest plot illustrating the association between right ventricular dysfunction and the risk of sudden cardiac death or ventricular arrhythmias among studies limited to patients with ischemic or nonischemic cardiomyopathy. In a subgroup analysis including four studies, RV dysfunction remained significantly associated with an increased risk of SCD or VA (OR 2.93; 95% CI 1.53–5.61), with moderate heterogeneity (*I*^2^ = 42%). RV, right ventricular; SCD, sudden cardiac death; VA, ventricular arrhythmia

During follow-up, 223 patients experienced the composite outcome of SCD or VA. RV dysfunction was significantly associated with a higher risk of SCD or VA (OR 3.69; 95% CI 2.40–5.66; *p* < 0.01), with moderate heterogeneity (*I*^2^ = 43%, *p* = 0.11), as shown in Fig. 2. As a supplementary analysis, we performed a subgroup analysis of studies assessing RV function by RVEF, with results consistent with the main analysis (Supplementary Figure S1). In a subgroup analysis restricted to studies enrolling patients with ischemic or nonischemic cardiomyopathy, the association remained significant (OR 2.93; 95% CI 1.53–5.61; *I*^2^ = 42%, *p* = 0.16) (Fig. 3).

Despite the limited number of studies, subgroup analyses by endpoint (SCD and ventricular arrhythmias) were performed. RV dysfunction was significantly associated with SCD in the three studies using SCD alone as the endpoint (OR 4.19, 95% CI 2.23–7.86) and with ventricular arrhythmias in the two studies using ventricular arrhythmias alone as the endpoint (OR 5.23, 95% CI 1.44–18.96), as shown in Supplementary Figures S2 and S3.

### Comparison of LV and RV function in predicting SCD and VA

Three studies (Chiba et al. 2024, Pandat et al. 2019, Malasana et al. 2011) provided both LV and RV function data in patients with and without SCD or VA, and in all three studies RV function was assessed using RVFAC. The studies by Chiba et al. (2024) and Malasana et al. (2011) included patients with ICDs implanted for primary or secondary prevention, whereas the study by Pandat et al. (2019) was a community-based registry.

**Fig. 4:**
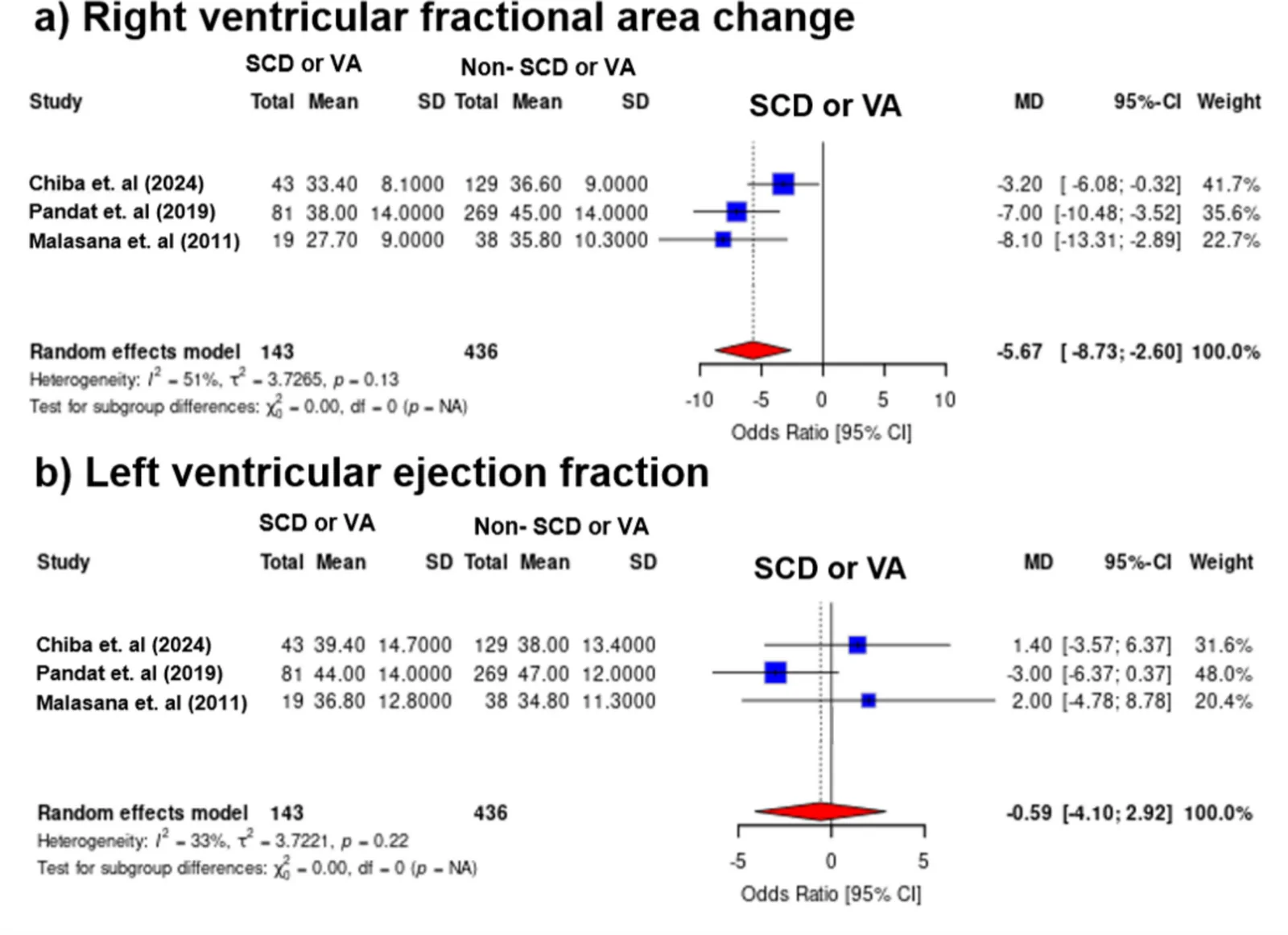
Forest plots illustrating comparisons of right ventricular (RV) fractional area change and left ventricular ejection fraction (LVEF) between patients with and without sudden cardiac death (SCD) or ventricular arrhythmias (VAs). **a** Sudden cardiac death and ventricular arrhythmias were related to low RVFAC. **b** In contrast, there was no significant difference in LVEF between the two groups. Patients with SCD or VA had significantly lower RVFAC compared to those without events, whereas no significant difference was observed in left ventricular ejection fraction between the two groups. SCD, sudden cardiac death; VA, ventricular arrhythmia

Using variance-based random-effects models, we compared the prognostic value of LVEF and RVFAC. LVEF did not significantly differ between patients with and without SCD or VA (MD − 0.59; 95% CI − 4.10 to 2.92; *p* = 0.74). In contrast, RVFAC was significantly lower in patients with SCD or VA (MD − 5.67; 95% CI − 8.73 to − 2.60; *p* < 0.01) (Fig. 4).

### Sensitivity analysis

Sensitivity analyses showed that the results were robust across all leave-one-out analyses. Notably, exclusion of Mikami et al. (2017) reduced heterogeneity substantially (*I*^2^ from ~ 49 to 4%), indicating this study was a major source of heterogeneity without altering the overall effect estimate.

## Discussion

In this meta-analysis of seven studies, we evaluated the association between RV function and the risk of SCD and severe VA. We also compared the prognostic value of RV function with that of LV function. To our knowledge, this is the first meta-analysis focusing specifically on RV function in relation to SCD and VA, offering new insights into risk stratification and potential indications for ICD therapy.

Our findings indicate (1) RV dysfunction is significantly associated with an increased risk of SCD and VA. (2) In both ICD cohorts and nonselective populations, a significant association was observed between RV dysfunction and SCD or VA, whereas a significant association for LVEF was not observed.

### RV dysfunction and prognosis

RV function has emerged as a critical determinant of prognosis in various cardiovascular conditions. Previous meta-analyses have demonstrated that RV dysfunction, assessed by cardiac MRI or echocardiography, predicts higher rates of mortality and hospitalization in patients with heart failure [22, 23]. Notably, Gorter et al. showed that RV dysfunction (based on tricuspid annular plane systolic excursion and RVFAC) was also associated with worse outcomes in patients with heart failure with preserved ejection fraction (HFpEF) [24].

These associations extend beyond traditional heart failure. RV dysfunction has been linked to adverse outcomes in myocarditis—including death, hospitalization, and arrhythmia [25]—and in pulmonary hypertension, where it is associated with increased risk of SCD and VA independently of LV function [26]. Similarly, in cardiac sarcoidosis, RV dysfunction has been tied to both VAs and SCD [27]. Collectively, these findings highlight the prognostic relevance of RV dysfunction across a range of cardiovascular diseases.

### Definition and assessment of RV dysfunction

In this meta-analysis, RV dysfunction was defined using several parameters, including RVFAC, RVEF, and RV strain, with differences across studies in imaging modality and cut-off values. This may represent a source of heterogeneity in the pooled estimates. Because of the complex geometry of the right ventricle, RV systolic function is difficult to capture with a single metric, and in clinical practice, it is commonly assessed using multiple parameters selected according to the underlying disease and pathophysiological context [28]. Although combining different parameters, modalities, and thresholds may have introduced heterogeneity, it may also reflect real-world clinical assessment of RV dysfunction. In addition, a study has shown that different RV indices derived from different modalities within the same population were similarly associated with ICD therapy, suggesting that the prognostic relevance of RV dysfunction may not be confined to a single parameter or modality [29]. However, the limited number of available studies precluded sufficiently powered subgroup analyses by parameter, modality, or cut-off value. Therefore, while our findings support the prognostic relevance of RV dysfunction, the optimal method for quantifying RV function for risk stratification of SCD and VA remains to be established.

### Mechanistic importance of RV function

The pathophysiological mechanisms linking RV dysfunction to adverse outcomes are increasingly recognized. While LV function has historically been emphasized, RV performance plays a key role in maintaining cardiac output by providing preload to the LV. RV dilation and interventricular dependence can impair LV filling [30], and combined RV systolic and biventricular diastolic dysfunction may reduce both cardiac output and coronary perfusion [31].

In HFpEF, approximately one-third of patients exhibit RV dysfunction [32]. These patients often show elevated pulmonary artery pressures, and progressive RVFAC decline has been linked to worsening pulmonary hypertension [32]. These observations suggest that worsening pulmonary hypertension may contribute to RV dysfunction through increased afterload, thereby reinforcing its prognostic significance. However, because RV dysfunction is also closely related to disease severity, pulmonary pressures, and LV function, residual confounding cannot be excluded.

### Clinical implications for SCD and VA risk stratification

This meta-analysis demonstrates that RV dysfunction is strongly associated with SCD and VA. Even in subgroups restricted to ischemic or nonischemic cardiomyopathy, this association remained robust. These findings suggest that RV dysfunction could serve as a valuable risk marker for identifying patients at high risk of life-threatening arrhythmias—particularly those who may benefit from ICD therapy.

Importantly, our secondary analysis suggested that RV function may provide additional prognostic information beyond LVEF for SCD and VA in ICD recipients (both primary and secondary prevention) and nonselective populations. The comparison between RV dysfunction and LV function should be interpreted in the context of the included study populations. Most included patients with established cardiac disease, often with already reduced LVEF. Therefore, our findings should not be interpreted as negating the established role of LVEF in current risk stratification but rather as suggesting that RV dysfunction may provide additional prognostic information beyond LVEF in selected populations. In addition, Xing et al. found that low RVEF was associated with fatal arrhythmic events in patients without structural heart disease [33], further supporting the prognostic value of RV function.

## Limitations

This study has several limitations. First, the definitions and assessments of RV dysfunction varied across the included studies, with different parameters and cut-off values used. This heterogeneity could affect the comparability of the results. In addition, the parameters included in this meta-analysis primarily reflected RV systolic function, whereas RV diastolic dysfunction was not evaluated. Although the clinical importance of RV diastolic dysfunction has increasingly been recognized, data regarding its association with VA and SCD remain limited, precluding assessment in the present study. Second, baseline patient characteristics varied among studies, which may introduce variability in the findings. The small number of studies available limited our ability to conduct more detailed subgroup analyses, and we focused on ischemic and nonischemic cardiomyopathy as a result. In addition, the small number of included studies also constrained the interpretation of publication bias assessments, including Egger’s regression test. Third, we were unable to separately analyze patients with ICDs based on primary versus secondary prevention indications, which could have provided additional insights. Finally, the comparison between LV and RV function was limited to LVEF and RVFAC, because no study provided both LVEF and RVEF data within the same patient population.

## Conclusion

Our meta-analysis demonstrates that RV dysfunction is significantly associated with an increased risk of SCD and VA. Furthermore, RV dysfunction may provide incremental prognostic information beyond LVEF in predicting these outcomes. These findings underscore the importance of incorporating RV function into risk stratification and clinical decision-making for patients at risk of SCD. However, given the variability in RV dysfunction definitions and the small number of studies included, further large-scale, well-designed studies are necessary to validate these findings, establish standardized assessment criteria for RV function, and better define its role in clinical practice, particularly for risk stratification and ICD therapy.

## Supplementary Information

Below is the link to the electronic supplementary material.

ESM 1 (DOCX 307 KB)

## Data Availability

No new individual-level data were generated in this study. The aggregate data underlying this meta-analysis were extracted from previously published studies cited in the article. The extracted data used for the analyses are available from the corresponding author upon reasonable request.
