# Supplementary material for: Right ventricular dysfunction: an overlooked predictor of sudden cardiac death and arrhythmic events—a meta-analysis
Source: Clin Res Cardiol. 2026 Jul 2;115(12):2216–26. doi: 10.1007/s00392-026-02976-6 (PMC13751778; doi:10.1007/s00392-026-02976-6)
Supplement: Supplementary file 1 [file 392_2026_2976_MOESM1_ESM.docx]

**Supplementary Material**

**Figure S1**

**Forest plot illustrating the association between right ventricular dysfunction assessed by right ventricular ejection fraction and the risk of** **sudden cardiac death or ventricular arrhythmia**

Low RVEF was significantly associated with an increased risk of sudden cardiac death or ventricular arrhythmia, with a pooled odds ratio (OR) of 3.42 (95% CI: 1.66–7.04).

RV, right ventricular; SCD, sudden cardiac death; VA, ventricular arrhythmia

**Figure S2**

**Forest plot illustrating the association between right ventricular dysfunction and the risk of sudden cardiac death**

RV dysfunction was significantly associated with an increased risk of sudden cardiac death, with a pooled odds ratio (OR) of 4.19 (95% CI: 2.23–7.86).

RV, right ventricular; SCD, sudden cardiac death

**Figure S3**

**Forest plot illustrating the association between right ventricular dysfunction and the risk of ventricular arrhythmia**

RV dysfunction was significantly associated with an increased risk of ventricular arrhythmia, with a pooled odds ratio (OR) of 5.23 (95% CI: 1.44–18.96).

RV, right ventricular; VA, ventricular arrhythmia
